# Bridging borders: shared strategies and challenges in abortion advocacy movements in Latin America and the United States

**DOI:** 10.3389/fgwh.2026.1852517

**Published:** 2026-09-23

**Authors:** Gabriela Alvarado, Paige Logan Prater, Elena Rodriguez Sanchez, Erin Leasure, Jessica Ogden

**Affiliations:** 1 International Center for Research on Women, Washington, DC, United States; 2 Ipas, Chapel Hill, NC, United States; 3 Department of Social and Behavioral Sciences, University of California San Francisco, San Francisco, CA, United States

**Keywords:** abortion, abortion policy, abortion rights, green wave, Marea Verde, reproductive justice, social movement

## Abstract

**Background:**

Feminist mobilization throughout Latin America has led a significant transformation in abortion policies, most widely known through the Marea Verde (Green Wave) movement. Concurrently, the United States has pushed abortion policies backward and this has accelerated following the 2022 Dobbs v. Jackson decision. Despite divergent legal and political trajectories, both regions face common challenges in abortion advocacy, shaped by distinct social movement traditions, political contexts, and histories of state-society relations.

**Methods:**

This qualitative study draws on 45 semi-structured interviews with activists, advocates, and experts in Argentina, Colombia, Mexico, and the United States. Guided by social movement theory, we conducted a comparative thematic analysis of interview data.

**Results:**

We identified ten key themes, organized within a model of social movement stages: problem recognition, proposed solutions, triggering events, openness to change, and resource mobilization. While context-specific strategies varied, the struggles and lessons learned were strikingly similar across geographies.

**Conclusions:**

Our findings challenge the notion that abortion advocacy movements are too contextually distinct for meaningful comparison. Instead, we demonstrate that shared challenges transcend national boundaries. We argue for the value of transnational dialogue and participatory research in strengthening abortion rights and reproductive justice movements. Policy and practice implications include investing in intersectional coalitions, prioritizing implementation, addressing processes of abortion-related stigmatization, and supporting grassroots efforts. Future research should expand comparative analyses and explore the evolving roles of digital and self-managed abortion activism.

## Introduction

Incremental and radical abortion policy changes in Latin America(LatAm) (1) have increased access to abortion across the region (2, 3), largely credited to the coordinated efforts of LatAm feminist groups and the Marea Verde (Green Wave) movement.

The Marea Verde gained prominence in Argentina in 2018, as activists organized nationwide vigils during a congressional debate on abortion (4). The green handkerchief, symbolizing the National Campaign for Legal, Safe, and Free Abortion, quickly became an emblem of the movement and spread across LatAm through media and social networks. Yet feminist mobilization in the region has a much longer history, influenced by political corruption and public distrust in government (5), with women's activism shaping peace processes, democratization, and human rights for decades (6–8). Central to this longer history has been the struggle against gender-based violence, including femicide, sexual violence, and reproductive coercion, which has been a galvanizing force for feminist and women's movements across Latin America and the Caribbean (5, 9). More recently, scholars have documented how LatAm feminist movements have developed distinctive strategies for abortion rights advocacy, including litigation, grassroots coalition-building, and community-based accompaniment practices that extend access beyond formal legal channels (10, 11).

The 2022 Supreme Court ruling of *Dobbs v. Jackson's Women's Health Organization* prompted activists and scholars throughout the United States to look beyond national borders for insights. Examining LatAm movements offers valuable lessons for understanding how grassroots activism can drive policy change in restrictive political contexts (12). A comparative analysis of abortion rights and reproductive justice (13) movements in the U.S. and LatAm can illuminate shared challenges and opportunities, reveal the conditions that promote or hinder advocacy efforts, and highlight lessons learned across movements.^1^ U.S. activists can draw from the advances achieved by LatAm activists, who have expanded abortion access despite strong opposition and deeply rooted religious traditions. Conversely, the U.S. experience following the Dobbs decision in 2022 provides important lessons for LatAm countries about the risks and realities of policy regressions amid a broader wave of conservative governance.

Several theorists have argued that certain features of social structure and context, such as inequitable capitalist structures, relative deprivation, or the availability of resources lead to the emergence of movements (14–16). Other scholars underscore the role of agency and leadership (17–20). More recent theories have shifted attention toward interpretive considerations, exploring how motivations, emotions, and individual decision-making processes shape participation in social movements (21). This turn toward agency emphasizes the diversity of movement participants and the ways in which their positionality, experiences, and identities influence both their engagement and the broader trajectory of collective action (17).

It bears noting that much of this literature draws on evidence and experience from the Global North and may fail to adequately capture the unique features of movements in the Global South. Scholars of LatAm social movements have long argued that the cultural politics enacted by movements in the region, including their struggles over citizenship, democracy, and collective identity, require analytical frameworks attentive to their specific historical and political contexts (22). In the analysis that follows, these social movement theories guide our exploration of abortion rights and reproductive justice movements in LatAm and the U.S. By applying these frameworks to case studies in Argentina, Mexico, Colombia, and extending them to the U.S., we aim to uncover the factors that have shaped the strategies, successes, and challenges of movements within their specific national and historical contexts. Our comparative approach is attentive to these dynamics: rather than treating the four study contexts as equivalent cases, we recognize that the relationships among them are shaped by histories of imperialism, epistemic coloniality, and unequal knowledge production. Drawing on decolonial feminist perspectives (23), we approach this comparison not as a search for universal lessons but as a transregional dialogue in which movements in each context have distinct epistemic authority over their own conditions and strategies.

To ground our understanding of abortion advocacy movements across the Americas, we drew on the writings of an array of social mobilization scholars including Blumer, Morris, and Smelser, to develop this definition: a social movement is a collective effort characterized by fluid and evolving membership, grounded in shared beliefs, that seeks to resist or transform the prevailing social order (15, 20, 24, 25).

The objective of this study was to understand the features of activism and movement building in different contexts and how they may have contributed to changes in the abortion rights and reproductive justice landscape. We see social movement research as a space for dialogue with and among communities where activists are engaged in the co-creation of knowledge through shared conceptualization, analysis, and theorization. By informing transregional dialogues, our analysis bridges social movement theory with movement building practice.

## Methods

In this qualitative study we present findings from a larger project, VITAL (Voices to Inspire Transnational Abortion Liberation). We conducted 45 virtual interviews with movement actors in Argentina, Colombia, Mexico, and the U.S. between November of 2024 and June of 2025.

### Sampling and recruitment

We selected four case study countries: Argentina, Colombia, Mexico, and the U.S. due to their history with feminist mobilization and/or the Marea Verde movement, their successes within the movement, and the diversity of historical contexts and legal mechanisms to secure abortion rights. Argentina is a classic example of strong and effective grassroots mobilization; Colombia illustrates judicial strategies and incremental reform; and Mexico is a federal system comparable to the U.S. where policy changes have focused on the state level rather than the federal level. Within Mexico, we collected data across three locations that reflect the country's subnational variation in abortion policy and social context: Mexico City (Distrito Federal), Hidalgo, and Chiapas. Mexico City has been a pioneer in abortion access since 2007, when it became the first jurisdiction in Mexico to legalize elective abortion in the first trimester. Hidalgo decriminalized abortion up to twelve weeks in 2021 and Chiapas decriminalized abortion in late 2024, which means we conducted interviews there in the months following this legal shift.

We conducted the U.S.-based research to explore the impact of federal policy rollbacks and specifically selected California, Georgia, and Texas to capture the breadth of the post-Dobbs U.S. policy landscape: California represents a state with strong legislative protections for abortion access and serves as a destination for out-of-state patients; Georgia represents a state with some access, subject to restrictive gestational limits; and Texas represents one of the most restrictive environments in the country.

In preparation for the project, we conducted six expert interviews with advocates and activists from LatAm and the U.S. to assess the feasibility and relevance of the proposed study. After obtaining feedback, we convened a Technical Advisory Group (TAG) of 16 experts across the four countries. The TAG worked alongside our team to develop interview questions, review data collection instruments, support participant recruitment, and assist in sense-making of findings.

Our team conducted a stakeholder mapping exercise to identify key actors in each location. We leveraged contacts through the TAG to reach out to individuals and invite them to participate and sent cold emails where we lacked contacts. Participants were offered a digital $50 VISA gift card for their time, or a directed donation in the same amount to an organization of their choice. Participants included scholars, legal experts, policy makers, healthcare workers, other nonclinical direct service providers such as abortion fund staff, activists, and advocates with expertise in the reproductive justice and/or abortion rights movements.

### Data collection

We obtained verbal consent from participants and conducted 45–60-min in-depth interviews. Four members of our team conducted interviews (GA and ER in Spanish; EL and PLP in English). We followed a semi-structured protocol, which explored five domains (Table 1).

**Table 1:** Semi-structured interview domains and sample questions.

Interview domain	Sample question
Movement participant characteristics	Could you tell me a little about yourself and how you became interested in abortion rights/reproductive justice? How did you come to work at your organization? What does your work look like?
Current abortion policy landscape	What do you think are some of the most pressing issues related to abortion where you live? In your opinion, what is your organization's place within the abortion rights/reproductive justice movement?
Movement success	Thinking about your own experience, what have been some of the conditions for success in your work?
Collaboration	Can you talk about how different organizations and actors work together in the abortion movement where you live?
Lessons learned	In your view, what is unique/special about the movement in your country and its achievements? What do you think are some lessons learned that might be helpful for others doing this work in other places? What do you want to get out of this research or learn from other advocates and activists in other places?

Additionally, we provided interview participants with an optional and anonymous demographic survey, administered separately after the interview concluded and alongside compensation. The survey was intentionally optional and delivered post-interview to avoid placing pressure on participants to disclose sensitive personal information, including lived experience with abortion, during or as a condition of the interview.

### Analysis

All interviews were recorded and transcribed verbatim for comparative thematic analysis. We used Dedoose (26) to facilitate qualitative coding. The lead author conducted a round of open *in-vivo* coding on the first four interviews conducted and recorded emergence of new codes in each new interview to assess for code saturation, assessing for the stabilization of the codebook structure rather than thematic saturation (27, 28). By the fourth interview no new codes emerged, the codebook was considered structurally stable, and codes were refined to construct a codebook through team consensus.

We employed a single codebook in English; transcripts were not translated at the analysis phase and were coded in their original language. The coding team (GA, ER, JO) completed training and proceeded to independent coding once substantial agreement (kappa > 0.6) (29) was reached through the inter-rater reliability feature on Dedoose. Discrepancies in coding were resolved by consensus in team meetings.

The research team met regularly to discuss emerging themes and drew from the coded data to construct themes using the matrix framework approach (30). We relied on social movement theory literature to shape the thematic organization using a combination of structural strain theory and an explanatory framework of social movement success (15, 25). Consistent with a descriptive-inferential approach to thematic analysis (31), our analysis aimed to produce themes that move beyond topical categorization to construct narratives grounded closely in participants' own language and meanings, without extending into interpretive theorization that would go beyond what participants explicitly described. This approach reflects our commitment to centering participant voices and avoiding the imposition of analytical frameworks onto the data.

### Ethical considerations

This study was reviewed and approved by the Institutional Review Board at the International Center for Research on Women. All methods and findings are reported according to the Standards for Reporting Qualitative Research (SRQR) (32).

Our team included researchers who are Latina, Asian, and white American women, feminists, moms, immigrants, daughters of immigrants, decolonialists, and reproductive justice advocates. Our backgrounds include abortion doula care, medicine, anthropology, sociology, public health, and public policy. We all collectively ascribe to the principles of reproductive justice and firmly believe in bodily autonomy. We are aligned with the organizations under which this research was conducted, firmly standing with experts, scholars, and activists in their commitment to safe abortion, accessible healthcare, and bodily autonomy. We acknowledge the role of emotion, motivation, and action in the study of social movements and draw from feminist theories in how we approach this work (33).

Understanding the complicated history of research, and “careers that are in large part parasitic upon the lives of movement activists” (34), we approached this work in the spirit of humility and constructive engagement. We sought to mitigate the risks of extractive practices by engaging in reflective introspection about our positionality as researchers and the power dynamics involved in research, as well as approaching this research through an equity-centered lens.

To strengthen rigor and transparency in data interpretation, we employed a two-phase participant validation process. In the first phase, participants who wished to engage further were invited to participate in structured discussion groups of five to six individuals, with representation across regions, using a deliberative democracy approach and visual scribing (35, 36). In these sessions, participants reviewed the constructed themes and exemplary quotes, and their discussion and feedback were used to refine and finalize the thematic analysis. In the second phase, all interview participants and TAG members received the final manuscript draft along with a structured feedback form designed to elicit substantive recommendations and editorial input, analogous to a peer review process. Feedback received was reviewed by the research team and incorporated prior to submission for publication. Participants also had the option to be included in the acknowledgements.

## Results

In Table 2 we present the distribution of interview participants by country/state and in Figure 1 by professional background.

**Table 2:** Interview participants by location.

Location	Number of participants
Latin America	**24**
*Argentina*	*7*
*Colombia*	*8*
*Mexico*	*9*
United States	**21**
*California*	*5*
*Georgia*	*6*
*Texas*	*10*

Bolded values represent regional totals.

**Figure 1:**
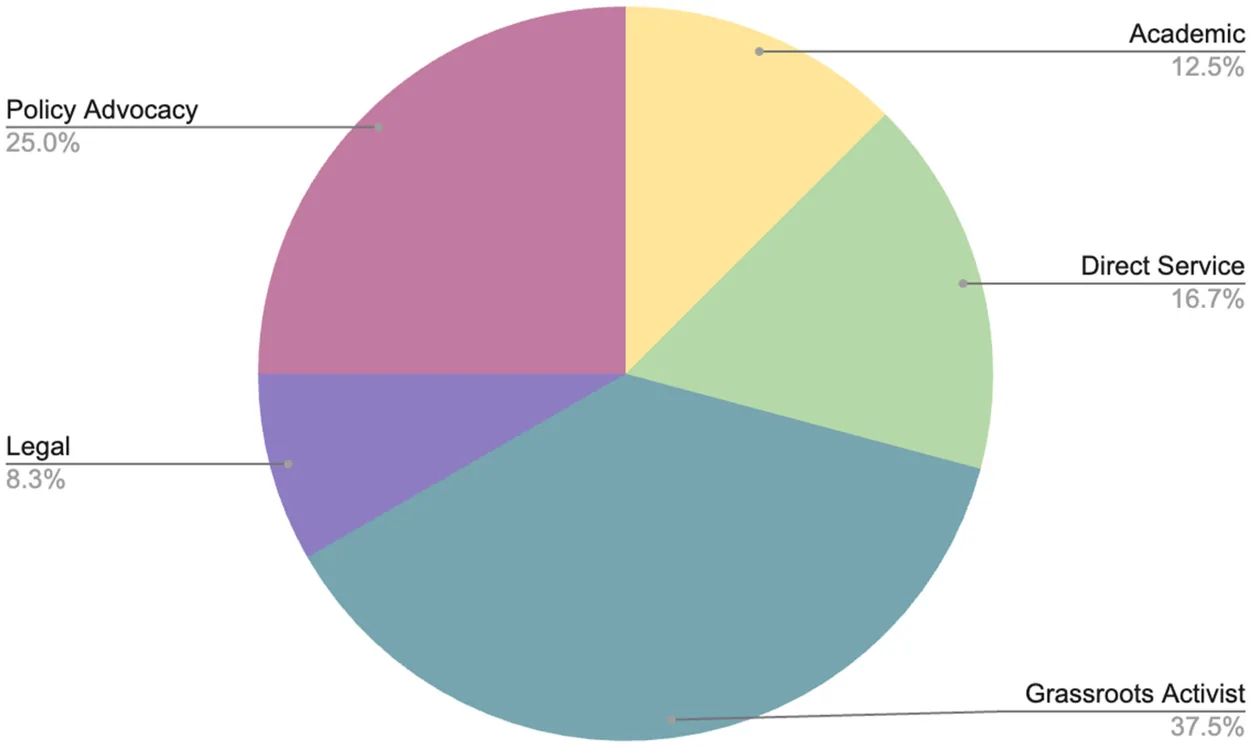
Interview participants by professional background (*N* = 45).

Of the 45 interviewees, 22 completed the optional demographic survey. Given the response rate represents less than half the sample, the demographic profile presented here may not be fully representative; however, as demographic characteristics were not used as analytic variables in our qualitative analysis, this gap does not affect the integrity of the thematic findings. In terms of race and ethnicity, participants described themselves as white, Black, Afro Indigenous, Latina, Mixed Race, Chicana, Mexican-American, Mestiza, Latin American, Hispanic, and Caucasian. The majority of the people we interviewed identified as women, between the ages of 26–55, lived in the country they were born, and were college-educated. Close to half (45%) of the participants who completed the survey shared that they had personal experience with abortion (Table 3).

**Table 3:** Interviewee demographic information, positionality, and lived experience.

Characteristic	Number of Interviewees (*N* = 22)
Age	
<25	1 (4.55%)
26–35	9 (40.91%)
36–45	3 (13.64%)
46–55	7 (31.82%)
<56	2 (9.09%)
Gender	
Woman	20 (90.90%)
Man	1 (4.55%)
Non-binary	1 (4.55%)
Do you live in a country where you were not born?	
Yes	3 (13.64%)
No	19 (86.36%)
What is the highest level of education you have obtained?	
Did not complete my bachelors degree	4 (18.18%)
Bachelors degree	10 (45.45%)
Masters degree	6 (27.27%)
Doctoral degree	2 (9.09%)
Do you have personal lived experience with abortion?	
Yes	10 (45.45%)
No	12 (54.55%)

In our qualitative analysis we identified ten themes which we organized in a model drawing from Smelser's and Nardini et al.'s work (Figure 2). The model outlines five stages within the abortion rights and reproductive justice movements following the problem and links them to qualitative themes.

**Figure 2:**
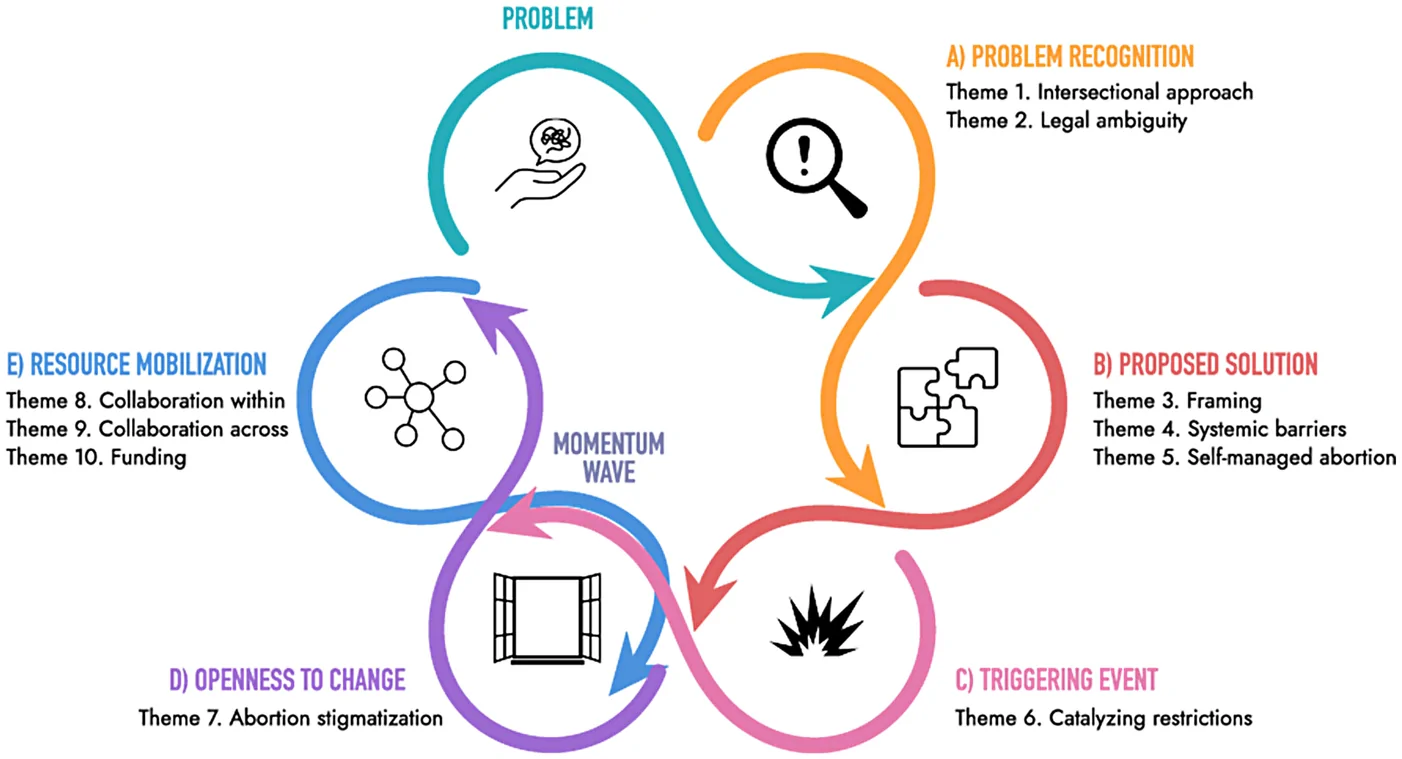
Social movement stages and corresponding interview themes.

Below we describe themes in detail (Table 4).

**Table 4:** Qualitative themes identified in interviews, organized by social movement stages.

Stage	Qualitative themes
(A) Problem recognition	*Theme 1*. Taking an intersectional approach creates a stronger foundation for social movements. *Theme 2*. The interpretation of abortion laws is key to abortion access; however, abortion laws are commonly misinterpreted.
(B) Proposed solution	*Theme 3*. Framing abortion as a central aspect of bodily autonomy and personal freedom helps create stronger ties to other social movements. *Theme 4*. Equitable abortion care requires addressing systemic and structural barriers. *Theme 5*. Self-managed abortion can expand access to abortion especially in restrictive and under-resourced environments.
(C) Triggering event	*Theme 6*. Increased restrictions in access to abortion can catalyze activism and policy responses.
(D) Openness to change	*Theme 7*. Abortion stigmatization exists on multiple levels, creating barriers to care and barriers to a unified reproductive justice movement.
(E) Resource mobilization	*Theme 8*. Collaboration within abortion rights and reproductive justice groups helps the movement grow, even when challenges arise. *Theme 9*. Collaboration across other social justice movements requires alignment of ideological positions, framing, capacity, and public support. *Theme 10*. Building a successful movement requires realignment of funding structures and priorities.

### Stage (A) problem recognition

Stemming from the underlying problem of inequitable and unevenly distributed access to abortion, the first stage of problem recognition is defined. This is where problems are identified and deconstructed. Here we included two themes: intersectional approaches and legal interpretations.

#### Theme 1. Taking an intersectional approach creates a stronger foundation for social movements

Advocates and activists shared that the movement for abortion rights and reproductive justice is deeply intertwined with other social issues, such as immigration, civil rights, gender equity, maternal mortality, disability, racism, and economic justice. They explained that individuals in the movement are increasingly recognizing the need for a more intersectional approach to movement building. This intersectional approach can be understood as “problem recognition”, because abortion rights and reproductive justice movement actors agree that there is more work to be done to frame health and social inequalities, including abortion access, through an intersectional lens.

Within the abortion rights and reproductive justice movement, activists and advocates occupy multiple social positions and hold multiple identities of varying privilege/disadvantage, often working across issues in personal or professional capacities. This has led to a call for greater acknowledgement of intersectionality within the movement, especially in light of the lack of diversity in dominant feminist movements.

“More and more groups are growing at the regional level, so they're also becoming more diverse, as new topics of discussion emerge on the sexual and reproductive rights agenda. We're also seeing a diversity of people, which has also helped decentralize that elitist, white, classist, heteronormative perspective.”—Colombia, Policy Advocate

Approaching abortion work through an intersectional lens requires reflexivity and humility. One interviewee shared their reflections on how their positionality impacts the work they do.

“I thought, how am I, as a white woman, going to participate in this social movement? I don’t want to impose from this privileged position, but I found out there was a way for me to combine my doctor side and my activist side in abortion care and advocacy—I can do both.”—Mexico, Direct Service Provider

By recognizing, acknowledging, and representing these intersections among storytelling, leadership, and activism, participants found that the abortion rights and reproductive justice movement can create bonds within their coalitions, break down barriers to collaboration, and create a stronger movement.

“[We need to] avoid fragmentation. I think that is fundamental, we are not going to be able to [do this] in solitude. I think that is also one of the lessons learned.”—Argentina, Grassroots Activist

Interviewees across geographies mentioned a range of intersecting power structures they were working within in their context—for example, the movement in Texas had a strong focus on immigration, in Georgia they emphasized connections with Black maternal mortality, and LatAm countries underscored gender-based violence and LGBTQI+ rights.

“My hope is as our focus kind of shifts to more reproductive justice, that we could continue to build additional partnerships. Because I really want to continue [working on] abortion access, but also the other stuff [..] We were supporting a bill that would give pregnant people access to clean water in prisons. So getting more into that aspect of reproductive justice”.—California, Policy Advocate

While participants broadly recognized the need for an intersectional approach, they admitted that complex organizing is challenging to do in ways that make sense to their communities.

“We need to call attention to the fact that the people we're trying to serve have more immediate and pressing issues than just repro, and we need to be able to frame reproductive justice as an issue that is an immediate need for them, so that they can understand the urgency.”—Georgia, Legal Expert

#### Theme 2. The interpretation of abortion laws is key to abortion access; however, abortion laws are commonly misinterpreted

Participants explained that abortion laws are often written ambiguously, which leaves room for interpretation that can be leveraged and weaponized by anti-abortion groups. This legal ambiguity, which was identified by interviewees as a core problem, is manifested and reenforced across sectors: within the healthcare system, legal system, and among the public.

When abortion laws are hard to interpret, healthcare providers may avoid providing the service or impose barriers for fear of prosecution. In California, for example, this issue has revolved around the term *viability*.

“We send about 20% of our callers out of state, because they are beyond 24 weeks. There’s a lot of conversation in California about viability [..] It is generally left up to providers, hospital systems, and medical systems to determine how they interpret the law, but it means that there aren’t nearly any providers who are providing care beyond 24–26 weeks here in the state.”—California, Grassroots Activist

In Georgia, the concept of *heart beat* introduces legal ambiguity (37).

“Miscarriage should not actually be affected by the word of the law. But we do see people get denied emergency care for miscarriage. You know the lines [are] very blurred [..] And even in instances where someone has an extremely wanted pregnancy but they experience a pregnancy loss, people are literally not receiving lifesaving care to the point where there have been multiple deaths that we know of in Georgia [among] people who did not receive timely emergency care.”—Georgia, Academic

In LatAm, where many laws allow for exceptions (“causales”^2^) to abortion bans “to preserve the health” of the mother, this also creates confusion, as people can interpret “health” differently.

“In Colombia [abortion] is completely decriminalized, up to the 24th week […] After the 24th week there are three ‘causales’ that if you understand them in a comprehensive way, there is no reason to deny an abortion.”—Colombia, Direct Service Provider

Further, in environments like the United States and Mexico, where abortion policies are frequently challenged, changing, and hard to follow, abortion providers and advocates must constantly adapt to changing legal frameworks.

“We realized that the legal context in Mexico became impressively complex. We have a lot of precedent from the courts, but also a lot of reforms. And at the same time these reforms are happening, they are completely different from one [state] to the next.”—Mexico, Legal Expert

If legal experts and health providers have difficulty interpreting laws, the public will not fare any better. Advocates and activists explained that when people have misconceptions about the law, then they will not seek out the service in the first place, which drastically reduces access.

### Stage (B) proposed solution

The second stage is the proposal of a solution that fits an ideological frame; here we included three themes: framing abortion as bodily autonomy and personal freedom, addressing systemic clinical barriers, and leveraging self-managed abortion.

#### Theme 3. Framing abortion as a central aspect of bodily autonomy and personal freedom helps create stronger ties to other social movements

Participants agreed that the abortion rights and reproductive justice spaces need to step away from mortality centered narratives. For decades, abortion rights advocates have centered their advocacy around the dangers of unsafe abortion, which is much more common when abortion is illegal. One activist noted that as self-managed abortion becomes more prevalent, this argument carries less weight.

“I mean we started out by talking of safe abortions to not die, to not stick coat hangers in ourselves, but now we are talking of other things. Like, you can have an illegal abortion now and not die.”—Mexico, Grassroots Activist

In exploring how interviewees used language, we found that LatAm advocates and activists tended to rely on a human rights framework more, while in the U.S. they leaned into the reproductive justice framework, which foregrounds social justice rather than what is interpreted as choice-based legal rights (38). One unique feature was that LatAm interview participants often shifted back and forth between reproductive rights and reproductive justice, using them interchangeably, whereas activists in the U.S. did not. Nonetheless, participants agreed that regardless of their guiding framework, they needed a powerful framing that would resonate with people outside the movement.

“We work from a full reproductive justice framework, believing in full bodily autonomy—the right to [have a] family or not [have a] family in the ways we choose. And we should be able to do that in safe, sustainable communities, and we should be able to do that free from over-policing, from the government.”—Texas, Direct Service Provider

Interviewees emphasized that linking abortion to bodily autonomy and freedom is a way to help other movements frame abortion rights within their own agendas. This framing aligns with social movement theory, positioning it as an ideological solution advanced by activists and advocates across both regions.

“Normalizing bodily autonomy, normalizing decisions that we're making for our bodies. I think it is something that could help [because] the fight for bodily autonomy is for everyone, not just a select few, not just women. But women, queer, trans, however you identify.”—Georgia, Grassroots Activist

Another activist explained that the notion of autonomy had the potential to link abortion with higher order ideals and depoliticize abortion. One activist explained that framing abortion around autonomy allowed the movement to build broad support by positioning it as a universal value rather than a divisive party-political issue.

“We found that the fight for autonomy is a political fight, but not a partisan fight. So what we defend is autonomy.”—Argentina, Grassroots Activist

A California activist shared that societies should “trust people in asserting their own bodily autonomy.” In the case of Colombia, this trust was operationalized in the Causa Justa court ruling.

“One of the novel things about the court ruling in Colombia was that in addition to the usual public health arguments of right to health, to dignity and equality, they affirmed the right to freedom of conscience. Meaning that abortion was not decriminalized because sometimes it has to happen or it is a last resort, but rather because your conscience tells you it is the right thing and people’s autonomy and agency allows them to make the right decisions [about their own bodies].”—Colombia, Direct Service Provider

Advocates and activists recognized that there was no one-size-fits-all approach to framing abortion. Instead, narrative frames that are grounded in historical and current political, social and cultural context have the best chance of resonating.

#### Theme 4. Equitable abortion care requires addressing systemic and structural barriers

Participants emphasized the difference between legality on paper and in practice. As one activist in Mexico stated, “decriminalization does not mean access”. For abortion to be legal *and* accessible, several things need to be in place: the underlying health system needs to be resilient and responsive to community needs, structural barriers to care must be addressed, and liberal abortion laws need to be in place and implemented with abortion seekers at the center.

Argentina, Colombia, and Mexico have universal healthcare, and despite system fragmentation, underfunding, and inequitable resource allocation, there is a core belief that health is a basic human right and a responsibility of the State.

“Colombia is a social State governed by the rule of law and the State has to recognize your rights. Not only does the State need to not interfere so that your rights are not infringed upon, but they need to guarantee and ensure that your rights are fulfilled.”—Colombia, Direct Service Provider

LatAm advocates highlight healthcare as a right, therefore free services is a central aim of abortion reform.

“[We demand] Legal abortion that is free of charge […] in a public hospital, or covered by social welfare.”—Argentina, Grassroots Activist

However, despite the aspirations for universal healthcare, in practice, large proportions of the population do not have access to care because they live in rural/underserved areas.

“It’s difficult because the northern region of Mexico is a desert. So there is a scarce population and between one place and the next you have to spend 8 h in a car where there is nothing other than desert.”—Mexico, Grassroots Activist

In the U.S. a combination of historical, political, and ideological conditions has led to a rejection of establishing health as a basic right, most evident by its privatized healthcare system. Interviewees from the U.S. brought attention to the idea of access to healthcare as a part of the broader concept of reproductive justice. One interviewee shared that despite one's stance on abortion, it is critical to make sure that people have access to as safe and healthy a life as possible, which can be supported by expanding Medicaid.

“That’s also compounded by the existing healthcare deserts. I know in Georgia at least, there’s a ton of rural counties that don't have a local hospital or don't have a local OBGYN and those people who are already negatively impacted by our societal systems and unable to get the general care they need are doubly unable to access abortion care if they have to travel out of state or go across state lines or miss work or miss childcare.”—Georgia, Legal Expert

One interviewee explained that there are areas that have historically experienced healthcare shortages even outside of abortion access.

“What’s not getting as much attention are the minority voices and people from underserved communities. And by that I would even include rural communities—places that [have] for a very long time been healthcare shortage areas. And the situation’s only gotten worse, not better, and not just with respect to abortion care.”—Texas, Direct Service Provider

Even places that are positioned as abortion access “havens” have challenges. For example, in California almost half of the counties do not have abortion providers.

“We're not actually a reproductive freedom state, because people have to travel a great distance within their home state, a state that supports access to abortion, to get basic healthcare.”—California, Grassroots Activist

Further, participants agreed there are systemic and structural barriers that impede access to all health services, not just abortion: racism, social inequality, housing, childcare, healthcare affordability, and supply chain issues. One interviewee explained that when it comes to abortion, given the time-sensitive nature and sociocultural complexity, all these systemic issues compound, decreasing access for marginalized communities.

“I think abortion care is like the canary in the coal mine. Like everything that is wrong with abortion care is actually wrong in society and wrong in medicine. And if we can fix abortion care, I think we will have a better world overall because it implies so many things.”—Mexico, Direct Service Provider

Activists and advocates in the U.S. drew from reproductive justice frameworks and intersectional perspectives to underscore how access to abortion is shaped further upstream at the system level rather than individual. Other barriers to access noted by interviewees included limited service hours in facilities, staff not being familiar with the legal right to abortion, and doctors not being familiar with abortion protocols.

For interviewees, it is not enough to legalize abortion; laws need to be operationalized into clinical protocols for implementation. Strategies to address implementation included preparing guidelines prior to legalization, training clinicians in post-abortion care, and providing clinicians a framework to interpret the law and protocols around viability and the definition of “risk to the health of the mother”. In Colombia, participants shared a strategy of capacity building for providers by leveraging post-abortion care training. Participants described strategies for building provider capacity in anticipation of legal change. One direct service provider explained how their organization used post-abortion care training to prepare clinicians for the moment the law changed.

“That’s exactly what we do. Post-abortion care so that when someone has that epiphany, we are ready to go.”—Colombia, Direct Service Provider

#### Theme 5. Self-managed abortion can expand access to abortion especially in restrictive and under-resourced environments

Many LatAm activists, particularly those in Mexico, proposed that abortion care is not wholly dependent on state infrastructure—people can be equipped to self-manage their abortions and this becomes a collective protest against medical hegemony. Further, where health systems have structural barriers such as low staffing, stigma, or supply issues, self-managed abortions can bridge the gap. Accordingly, self-managed abortion arose as a third solution to strengthen and advance the abortion rights and reproductive justice agenda.

“We have to depathologize and remove medical hegemony, because a person who needs an abortion and has pills in their hand, they have a lot of power.”—Argentina, Grassroots Activist

Interviewees explained that when people are provided information and resources for self-managed abortion, they can make informed choices about their reproductive health despite structural or systemic barriers to procedural abortions.

“That same OBGYN [..] taught me how to manage abortions with medications at home. When I discovered that we didn’t need doctors, I was like, this is where it takes off. And that is where our model for safe abortion accompaniment at home started, using the WHO protocols.”—Mexico, Grassroots Activist

One direct service provider in Mexico shared how they came to the realization that “doctors are basically not that important in abortion care”. Another provider underscored that self-managed abortions were not only approaches that offered autonomy to people but were also safe and effective.

“I think there are many ways to provide access, and not just from the medicalized perspective. There are also accompaniment models and other effective and safe strategies that can provide people with human dignity.”—Colombia, Direct Service Provider

However, interviewees had mixed opinions about self-managed as a potential solution. They acknowledged the role of self-managed abortion in contexts with restrictive laws and access; however, they warranted caution. One activist emphasized, “if the state has to implement, then they should do it well,” meaning that self-managed abortions let the government off the hook in their responsibility to provide services. Activists and advocates explained that there are a range of reasons why individuals may prefer a procedural abortion. Another interviewee added that over emphasizing self-managed abortion may unintentionally relegate abortion as something to cover up, potentially fueling abortion stigmatization.

Activists in Mexico explained the versatility of accompaniment models that have been instated to support self-managed abortion, including supporting people across borders through virtual encounters, and an expanded role in civil society oversight in states where abortion was legalized to ensure the state was fulfilling its obligations to provide guaranteed services.

“So what we do is a surveillance and monitoring of abortion services through the Health Department. So when people reach out to us, we tell them that it’s important that they know that it is their right to access a safe and free abortion through the public health system. And so we give them the information and tell them we can accompany them in any of the two ways; be it self-managed at home with us, or through the health system which ends up being at home since they only provide the medication.”—Mexico, Grassroots Activist

U.S. activists and advocates discussed how self-managed abortion can reduce costs and increase access, particularly for people who experience discrimination in healthcare settings. One policy advocate shared how they think of self-managed abortion as just one piece of the puzzle.

“I'm trying to find the balance between all of the scenarios, and just prepare myself mentally, while not necessarily putting all of my eggs in one basket. Because things might come up that I don't know, there are so many horrific things that [the administration] can do that if we put all of our eggs into medication abortion, for example, then we might get distracted from abortion surveillance (the monitoring of abortion statistics).”—California, Policy Advocate

This theme reveals a conceptual and strategic difference between the regions: while in LatAm self-managed abortion is seen as both as an emancipatory and potentially limiting practice, in the U.S. it is perceived predominantly as another pathway to increase access to care.

### Stage (C) triggering event

The third stage is the occurrence of a triggering event, under which we included one theme: how restrictions can catalyze efforts. This triggering event leads to governments and societies being more open to change, which comprises the next stage.

#### Theme 6. Increased restrictions in access to abortion can catalyze activism and policy responses

Participants shared examples of how events can focus attention within the movement, which aligns with the social movement stage of “triggering events”. In talking with participants we found that abortion restrictions seemed to act as a focusing event that catalyzed responses.

In Colombia, prior to legalization, barriers to access continued to coalesce, making it clear that the legal framework at the time (three *causales*) was insufficient. For example, medical boards were instated to determine individual cases, and providers were given the right to conscientious objection.

“Sadly, many barriers were consolidating against access and we felt there were too many limitations to the three causales [..] In 2020 we had several meetings and we said ‘this has reached the limit’.”—Colombia, Policy Advocate

It is worth noting that Colombia's broader legal trajectory has moved in a liberalizing direction over time, culminating in full decriminalization in 2022; however, participants described the practical limitations of the causales framework not as a regression but as a gap between formal legal protections and lived access, the closing of which they experienced as an urgent catalyst for action.

Publicized cases of injustice related to abortion, such as Black maternal mortality in the U.S. or incarceration in LatAm, also catalyzed political backlash and demands for protections. In Georgia, cases of Black maternal mortality provided an impetus for mobilization among typically sidelined communities.

“A lot of black women finally saw themselves in this abortion advocacy work because they saw how familiar Amber [Thurman] and Candi [Miller] looked.^3^ Like these are like our cousins, our sisters, our friends, you know, so it really connected folks. So at that rally, we had people who'd never been to a rally before.”—Georgia, Grassroots Activist

One interviewee from Argentina explained how she realized the movement was gaining momentum when she ran out of the green handkerchiefs (symbol of the *Marea Verde*) she had been storing in her home for ages.

“I think that opened a door, and we jumped head first. We saw the opportunity and we had the artillery ready, and I think that’s when we knew we were close—when we were marching alongside people we didn’t know.”—Argentina, Policy Advocate

Restrictions did not necessarily have to occur in the same location for activists and advocates to be catalyzed into action. For example, SB8 in Texas (39), known as the Texas Heartbeat Act, which banned abortion after approximately six weeks of pregnancy and empowered private citizens to sue anyone who aided or abetted an abortion, prompted action in California, encouraging advocates to work towards making abortion and contraception constitutionally protected in the state.

“The trigger to all of this was SB8 in Texas, which was the bounty hunter law as you know. We quickly came into action, and we created this partnership with the governor, created this partnership with the Attorney General’s Office, worked with the legislature, and just put together a report with policy recommendations. It involved the work of more than 200 people and 40 organizations.”—California, Grassroots Activist

Support networks, mutual funds, and transnational collaboration have also been catalyzed by restrictive laws. One organization explained how in the face of restrictions, they just shifted their resources.

“I think one of the most successful collaborations we've had was after Dobbs, when we were paused for nine months, and for some of that time, we partnered with a practical support organization that supports people who live in or travel to the Midwest region. And at the time we were waiting for some of the litigation to clarify what we could and couldn't do in Texas. So we worked with them to support some of their clients who were from legal states, traveling either within their own state or to another legal state and freed up some of their capacity.”—Texas, Direct Service Provider

Far from halting movements, adversity seemed to strengthen internal cohesion and accelerated tactical innovation.

### Stage (D) openness to change

Under this fourth stage we included the theme on abortion stigmatization. The more open societies are to change, the easier it is to mobilize resources, which is the final stage. When resources are adequately mobilized, this allows the movement to gain traction, looping back to this stage and generating even more openness to change. More openness to change then also increases the number of resources available for mobilization, and this reinforcing loop continues until the movement is successful in achieving its goal. We have labeled this reinforcing process the Momentum Wave.

#### Theme 7. Abortion stigmatization exists on multiple levels, creating barriers to care and barriers to a unified reproductive justice movement

Abortion stigmatization has a profound impact on public support for abortion. While stigmatization manifests across the social movement cycle, it is undeniably apparent in the reinforcing cycle of the Momentum Wave. Activists discussed the complexity and multi-level nature of abortion stigmatization: within communities, within and between social movements, and throughout the medical system. They emphasized how heavily stigmatized abortion is, which leads to shame and social (sometimes criminal) punishment. Even in settings where abortion is legal, abortion seekers are highly cognizant of social repercussions.

“You can do whatever you want with policy. You can make abortion legal in every corner of this country, but until peoples’ attitudes change, until there’s a culture shift, things won't change.”—California, Grassroots Activist

One activist further explained the links between stigmatization and individual outcomes.

“I'm talking to people who are supportive of abortion but still have stigmatizing beliefs and attitudes about abortion. And we know that stigma and misinformation lead to poor outcomes for people who are seeking abortion care. We know that more perceived stigma is associated with more emotional distress and feelings of isolation.”—Georgia, Academic

Several participants also shared that in many contexts, religious organizations contribute to the widespread perpetuation of shame-based narratives.

“Latin America is a region strongly influenced by religion, especially Catholicism, and now Evangelicalism is making significant progress. But through these religious narratives, we are also contributing to cultural change, that is, we are beginning to dismantle these stories and narratives that precisely create obstacles to the exercise of rights.”—Colombia, Policy Advocate

Activists in the U.S. are also working with communities to reinterpret faith-based narratives to explain that reproductive justice values are aligned with values that religious organizations promote.

“These values that I was taught in church and that I was taught at home, they are the same values that we should experience as human beings when we talk about how important it is that each person should decide about their body; no one should interfere with them.”—Texas, Grassroots Activist

Interviewees also noted the presence of stigmatization within the abortion rights and reproductive justice movement and that they have had to put in the work to address these biases and misconceptions. For example, one Argentinian activist explained how their campaign slogan, “sexual education to decide, birth control to not abort, and legal abortion to not die”, was problematic.

“We said, ‘enough with saying birth control to not abort’, abortions are not the issue. We needed to remove the layers of prejudice amongst ourselves.”—Argentina, Grassroots Organizer

Lastly, interviewees emphasized the role of the medical system, including providers, in promoting abortion stigmatization. Notably, stigmatization in the form of conscientious objection by clinicians severely impacts access to care. One interviewee shared how abortion policy changes have started to increase the levels of societal acceptance of abortion, given that it is now a service provided by the public health system. Nonetheless, they receive notice of cases of service denials where they need to advocate for individual people to receive the service they are legally entitled to.

“Because even though this right was in [blinded Mexican state] and the whole country, no one ever accessed the service because providers would impose their own beliefs, ‘no, you’re going to die’, ‘no, this is wrong’.”—Mexico, Grassroots Activist

Overall, abortion stigmatization persists as a cross-cutting barrier that limits both policy implementation as well as coalescence of the movement.

### Stage (E) resource mobilization

Under the final resource mobilization stage, there are many components, including building grassroots momentum, assembling networks and coalitions, and maintaining a leaderful approach to the movement. In this stage we included the following themes: collaborating within and across movements, and funding prioritization.

#### Theme 8. Collaboration within abortion rights and reproductive justice groups helps the movement grow, even when challenges arise

Collaboration was widely recognized by activists and advocates as a cornerstone of effective movements. Across contexts, participants described how coalitions, partnerships, and intergenerational alliances have enabled organizations to pool resources, amplify marginalized voices, and respond rapidly to shifting political landscapes. Interviewees highlighted successful examples of collective action, such as coordinated responses to restrictive legislation, mutual aid networks, and the sharing of best practices across borders and generations.

“The day SB8 went into effect, [we were] like all right, who do we call for this? Who do we call for that? We already knew all that as other states started to pass similar laws or trigger bans went into effect. Those organizations didn't have to reinvent the wheel [..] Just being able to lean on each other is a big success.”—Texas, Direct Service Provider

For collaboration to be effective, there needs to be trust, which takes time. There needs to be trust between communities and organizations, across organizations, and within organizations.

“I don’t think the political trust appears from one day to the next, you build it and this also goes with the national and local organizations, with researchers, and activists.”—Argentina, Academic

Collaboration was a central discussion topic in all interviews, yet it was described differently across countries. In Argentina there was a hierarchical approach to collaboration where individual organizations would affiliate themselves under a national campaign—a formal, unified structure in which individual organizations affiliate under a shared platform and common set of demands, as exemplified by the National Campaign for Legal, Safe, and Free Abortion. In states like California and Georgia there are statewide coalition efforts—looser alliances of organizations that coordinate around shared goals while maintaining organizational autonomy and attempting to minimize hierarchy. On the other hand, Mexican activists prioritized coordination—an even more decentralized approach focused on moving in “the same direction” without requiring close institutional collaboration or formal affiliation, allowing diverse groups to act in parallel without merging their agendas.

One often overlooked form of collaboration that was highlighted by interviewees in both the U.S. and LatAm was intergenerational learning. Participants highlighted the wisdom and resilience of older activists who have endured past struggles, and the creativity, vision, and energy that young people bring to the movement.

“You can't get anywhere [..] without knowing where you came from and what happened in the past. And I think that it’s really important to acknowledge that, and lean into intergenerational connections when we can”—California, Grassroots Activist

Despite the desire for collaboration, participants explained that abortion rights and reproductive justice movements across the region have internal tensions stemming from funding competition, fragmented strategies, and historical inequities. Participants shared that larger, well-resourced organizations can often overshadow grassroots groups, leading to mistrust. Funders' preference for high-profile, short-term initiatives exacerbates these dynamics.

“I think there’s been this huge disconnect between [what is prioritized at the national level and] what is needed at the local level, what is feasible at the local level, what makes sense at the local level, what people want at the local level [..]. I do think it’s important to talk with each other about what may work, and collaborate with different States, but not that we try to turn everything into one big national campaign that’s led by folks who don't know what it’s like to try to get an abortion in Georgia.”—Georgia, Academic

Overall, while collaboration was recognized as a crucial element of the diverse social movements for abortion rights and reproductive justice, many participants emphasized that it alone cannot resolve the multifaceted barriers to access.

#### Theme 9. Collaboration across other social justice movements requires alignment of ideological positions, framing, capacity, and public support

Advocates and activists explained that engaging with allies in other social justice movements was an important part of resource mobilization.

“We have allies not only in reproductive justice, but also in economic justice, and we have gone with them to march, to speak out so that people are paid, because here in the Valley many companies don't pay people because they don't have documents, so we have to be united. I also think that the key is to unite with other organizations, with our allies, and that their needs or their struggles are our struggles.”—Texas, Policy Advocate

In general, interviewees emphasized that abortion rights and reproductive justice organizations support other issues such as immigration, racism, and equity because social justice is indivisible.

“By bringing the [reproductive justice] conversation to environmental justice orgs and criminal advocacy orgs, we’ve been able to create what I call a coalition here because we work with so many other orgs that call us in to either have us do [reproductive justice] training or just to connect reproductive justice to whatever injustice that they're dealing with.”—Georgia, Grassroots Activist

Advocates and activists shared several barriers to establishing successful, genuine, and productive cross-movement collaborations, including ideological alignment. Interviewees explained that other social justice movements did not always understand how abortion rights and reproductive justice fit into their causes. For example, one participant from Argentina explained the challenges in collaborating with male-dominated workers unions.

“There is a structure that unions have that helps a lot in getting organized, but it has the challenge that still the majority of those union authorities are male. So we have to elbow our way in to claim space.”—Argentina, Grassroots Activist

Another issue participants mentioned was the overall bandwidth of other movements. Social justice movements have widespread capacity issues, underfunding, and burnout, which means that collaborating across causes has the potential to place strain on individuals and organizations. One participant explained the issue of risk tolerance when it came to public support for abortion rights.

“So everybody cannot, for various reasons of risk tolerance and everything, [they] can’t show up in the same ways for abortion that they can for their own issue. There’s a political cost sometimes for our partners who believe in us, who are aligned with us and our values.”—Georgia, Grassroots Activist

While some interviewees understood these trade-offs, others were more frustrated by the perceived lack of reciprocity.

“I can tell you that not all human rights organizations at the national level are with us on this fight for abortion, and we also sometimes get hung up on that.”—Mexico, Legal Expert

One participant reflected that peers in the abortion rights and reproductive justice movement have a tendency to be selfish, which is important to address when working towards collective agendas.

“I believe that the agendas of freedom, of equality, are collective agendas and that all of us need to weave together unity and solidarity between organizations. That for me is the biggest advice [..] we have to situate the agenda of sexual and reproductive rights on the agenda of democracy because we have to be [on the same page], because we are very selfish. In other words, we want everyone to join our cause and support us in our cause [but we don’t always reciprocate].”—Colombia, Policy Advocate

Despite the difficulties in operationalizing cross-cutting social justice movements, advocates and activists emphasized that it is necessary and important.

#### Theme 10. Building a successful movement requires realignment of funding structures and priorities

Effective movement building relies on targeted resource mobilization that supports and uplifts community priorities. The distribution of financial resources was an important discussion for participants in all countries. Many noted that national-level organizations receive financial support at the expense of work done by local organizations.

“One critique that has emerged by those of us who work in accompaniment collectives is regarding the large organizations. At the end of the day they receive a gush of money and they end up reporting on the projects that are done by us in the collectives, but without paying us anything.”—Mexico, Grassroots Activist

One interviewee in Texas echoed the sentiment on redistributing resources for a fairer allocation that takes into consideration the work that is done “on the ground”.

“In the US, this tension is holding us back: we need to drastically shift the priorities of our movement away from this grand investment in [national-level] policy advocacy that leads to these splintered campaigns that actually end up going nowhere because they're more for brand recognition and donor dollars than the actual end goal. We need to invest first in abortion funds and direct service work.”—Texas, Direct Service Provider

As participants reflected on the challenges of financial resources, they explained that lack of funding places a lot of pressure on volunteers who are committed to supporting the movement, often at the expense of their personal capacity and well-being.

“Those funds come to us intermittently, so we try to recognize those who have put in most work into projects. We do try with these funds to cover meals, transportation because there are [..] people who are unemployed or university students and since we are all here voluntarily we try to distribute in the most equitable manner.”—Colombia, Grassroots Activist

Another participant shared thoughts on how they feel that funders inadvertently create a system that does not foster collaboration within and across movements.

“Because right now it’s harder to collaborate. When you have very individualistic goals they are driven by grants, like I have to do this thing because this foundation told me I have to do it and I don't have time to build with my peers towards our shared vision for Georgia because I got to do this thing or I'm going to have to give the money back or I'm not going to get the money again.”—Georgia, Grassroots Activist

Lastly, several advocates and activists raised concerns about donors overlooking well-established abortion funds in the U.S. and accompaniment collaboratives in LatAm in favor of new and “flashy” projects.

“What I’m seeing a lot from the movement at large is every year we create something new, which feels like a brand new thing, but it’s actually a copy [..] And so, we spend all these resources for the new and shiny thing instead of supporting the thing that’s existing. And so I think that breaks our role in terms of having sustained coalition work.”—California, Policy Advocate

Taken together, these reflections reveal that sustainability of the movement requires a transformation of funding models, supporting long-term investments and strengthening local commitments.

## Discussion

This study explored the features of abortion rights and reproductive justice movement building through interviews with 45 advocates and activists in Argentina, Colombia, Mexico, and the U.S. We organized the themes along a model of stages which included problem recognition, proposed solutions, triggering events, and then a two-part reinforcing loop Momentum Wave that was composed of openness to change and resource mobilization.

When we embarked on this project we wondered whether it was feasible to compare social movements in different geographies with unique sociopolitical contexts. We understood that social movement building does not follow a specific recipe for success, nonetheless, we acknowledged the possibility that the abortion rights and reproductive justice movements in Argentina, Colombia, Mexico and the U.S. could be too different for any meaningful comparative analysis. However, this was not the case. Instead, we found an overwhelming number of commonalities across the stories shared in the four countries.

While we found obvious differences between the countries' sociopolitical contexts, history, and specific movement tactics, their struggles were aligned. Challenges that were shared by activists in one context and thought to be unique to their context, were almost always mirrored in the other countries. The most prevalent theme across geographies was the recognition of the problem through an intersectional lens.

LatAm scholars have demonstrated that abortion is intertwined with racial inequality (40). Critical race and intersectionality scholars in the U.S. such as Kimberlé Crenshaw and Patricia Hill Collins have further described how overlapping identities and social locations shape distinct experiences of discrimination, oppression, and privilege (41–44). Applying this perspective within the abortion rights and reproductive justice movement requires critically examining who constitutes the movement's leadership and constituency, and whether its agendas reflect the movement's heterogeneity, in alignment with Rita Segato's decolonial perspectives, which caution against overreliance on state reforms and emphasize the agency of Indigenous and marginalized communities (23). Verónica Gago's theorization of the feminist strike as a form of collective action that traverses and connects diverse sites of struggle similarly illuminates how LatAm feminist movements have built transversal power across differences of class, race, and geography, offering a framework for understanding the coalitional dynamics our participants described (9). Interviewees demonstrated a high degree of self-reflexivity on these issues, illuminating how multiple, mutually reinforcing structural dimensions of power infiltrate the everyday experiences of abortion seekers and advocates.

Yet our findings also surface a productive tension in which participants broadly embraced intersectional framing as theoretically and politically compelling, yet many simultaneously described the practical organizing challenges it creates. Expanding the movement's agenda to encompass immigration, racial justice, housing, and economic equity can deepen coalition breadth but also diffuse focus, strain organizational capacity, and complicate messaging for communities whose most immediate concerns lie elsewhere. This tension between the inclusivity that intersectionality demands and the strategic focus that effective organizing often requires is not unique to abortion movements, but it is particularly acute in a landscape where political windows are narrow and resources are scarce. It is also worth noting that the concept of autonomy invoked by participants across contexts may reflect distinct political and theoretical traditions; feminist philosophers have distinguished relational autonomy from more individualistic liberal conceptions, and this distinction maps onto differences observable in how participants across our study settings framed bodily autonomy, with LatAm participants often emphasizing collective struggle and structural conditions alongside individual rights.

Although intersectionality is widely used among contemporary social movements, the concept is relatively new in social movement scholarship. According to Roth (2021), structural intersectionality focuses on how individuals within a movement experience their own social location and status in terms of gender, class, race, age, or sexual orientation, while political intersectionality looks at how the positionality of the overall group shapes the collective identity being constructed (44). Interview participants were well aware of these dynamics and pointed to collaboration and coalition building as strategies in the pursuit of a truly intersectional movement.

This study makes unique contributions to the literature on social movements and abortion advocacy research. First, by employing a comparative qualitative approach across four countries, we illuminate both shared and divergent strategies in abortion advocacy, challenging the assumption that movement dynamics are too different to compare across contexts. Second, our integration of intersectionality and social movement theory provides a nuanced framework for understanding how identity, resource mobilization, and political opportunity interact in shaping movement efforts and outcomes. This framework is helpful given that feminist activism does not work only in antagonism to the state, with feminists who work within state institutions playing a pivotal role when they act as intermediaries between civil society and government, as shown in Mexico, where *femocrats* have been instrumental in defending abortion rights by bridging state and movement networks (45). Third, our reflexive methodology foregrounds the voices of activists and advocates, offering practical insights for movement-building that are often absent from academic analyses.

A further conceptual contribution of this study is the Momentum Wave, a reinforcing feedback loop in which openness to change and resource mobilization mutually amplify one another, generating the conditions for sustained movement growth. This concept is aligned with existing dynamic frameworks in social movement theory. Tarrow's concept of “cycles of contention” describes how one movement's success creates political opportunities that enable other movements to act, producing cascading waves of collective action across a political system (46). McAdam's political process model similarly identifies cognitive liberation as a key precondition for mobilization, emerging from the interaction of political opportunities and organizational strength (47). These frameworks are foundational, yet they tend to treat their constituent elements as sequential inputs or additive preconditions rather than as dynamically self-reinforcing processes occurring within a movement over time. The Momentum Wave extends this literature by proposing that openness to change and resource mobilization do not simply co-occur but actively produce one another in an ongoing loop: as public attitudes shift, new resources become available; as resources accumulate, movements gain the capacity to further shift attitudes.

While the advocates and activists who shared their stories with us noted that there was no single pathway to success, they were excited to share lessons learned in hopes of strengthening solidarity for abortion rights and reproductive justice that defies national borders. Nonetheless, we avoid making generalizations here on the strategies and approaches shared by interview participants and recognize that approaches that are effective in one place in time may be counterproductive somewhere else or in the same place at a different time (34). Given this, we recognize the generalizability of these lessons are contextual and dynamic. Lastly, we acknowledge the methodological limitations such as coding approach, analytic strategies, sample size and potential participant-researcher alignment. Code saturation was reached after four interviews, which is consistent with the *in-vivo* codebook development approach used in this study; however, it is possible that additional codes could have emerged from a larger or more diverse initial coding sample. Nonetheless we mitigated bias through reflexive discussions, TAG input, and sharing findings with participants. Lastly, our thematic analysis was conducted at a descriptive-inferential level (31), staying close to participants' own language and meanings rather than extending into interpretive theorization; while this approach centers participant voices and minimizes the risk of imposing external frameworks onto the data, future work employing interpretive thematic analysis could probe more deeply into the theoretical traditions and structural logics embedded in these movements across contexts.

Our findings have several implications for policy and practice. For activists, we underscore the importance of intersectional coalition-building through storytelling, solidarity, and collective care (48), and the need to address internal tensions around resource allocation and strategy. The reflections from our participants on self-managed abortion and accompaniment connect to scholarship documenting how LatAm feminist accompaniment networks conceptualize self-managed abortion not merely as an access strategy but as a form of feminist political praxis that challenges medical hegemony and enacts collective autonomy (49, 50).

Policymakers should recognize that legal reforms alone are insufficient; attention must be paid to implementation, education, and stigmatization, as evidenced in Colombia and across LatAm, where conservative legal counter-mobilization and procedural challenges continue to threaten access even after formal legal victories (12).

Funders and movement leaders should prioritize sustained investment in grassroots and intergenerational efforts. For researchers, future studies should explore the trajectory of self-managed abortion advocacy, the role of digital activism, and the transferability of movement strategies across contexts. Comparative research should also consider expanding to additional countries and subnational regions to further explore social movement stages and dynamics in other contexts.

## Conclusions

This study advances the field by providing a comparative, transnational analysis of abortion rights and reproductive justice movements, highlighting both common challenges and context-specific strategies. By centering intersectionality and reflexivity, we offer a framework that bridges theory and practice, and foregrounds activist expertise. However, our findings should be interpreted in light of the inherent challenges of cross-context comparison. Our results suggest that activists should invest in intersectional coalition-building, that policymakers focus on implementation and stigma reduction, and that funders support grassroots and intergenerational efforts. Future studies should build on this work by examining additional contexts and exploring the evolving role of digital and self-managed abortion activism.

## Data Availability

The datasets presented in this article are not readily available because Sensitive individual interview data will not be shared, per our IRB approval. Requests to access the datasets should be directed to jogden@icrw.org.

## Footnotes

^1^Here we will use the term *abortion rights and reproductive justice movement* as an overarching term to recognize the preferred terminology among activists and advocates in each of our regions of study. Nonetheless, we recognize that these terms cannot be conflated, are context dependent, and have different connotations and histories.

^2^The three causales refers to the exception model where abortion is allowed in the case of three specific causes or exceptions: when the pregnancy was the result of rape or incest, there are severe fetal malformations, or imminent risk of death to the pregnant person.

^3^Amber Thurman and Candi Miller were two Black women in Georgia who died in 2024 after being denied timely abortion care following the state’s restrictive post-Dobbs legislation; their cases received widespread national attention and galvanized abortion rights activism, particularly among Black communities. The rally referenced here was held in response to their deaths.
