# Correction: Bridging borders: shared strategies and challenges in abortion advocacy movements in Latin America and the United States

**DOI:** 10.3389/fgwh.2026.2008504

**Published:** 2026-10-06

**Authors:** Gabriela Alvarado, Paige Logan Prater, Elena Rodriguez Sanchez, Erin Leasure, Jessica Ogden

**Affiliations:** 1 International Center for Research on Women, Washington, DC, United States; 2 Ipas, Chapel Hill, NC, United States; 3 Department of Social and Behavioral Sciences, University of California San Francisco, San Francisco, CA, United States

**Keywords:** abortion, abortion policy, abortion rights, green wave, Marea Verde, reproductive justice, social movement

In the published article, the acknowledgments section was missing. The statement that should appear reads:

“We would like to thank the members of our technical advisory group as well as the individual participants who shared their stories with us, including Verónica Cruz (Las Libres), Nayla Luz Vacarezza (Universidad de Buenos Aires), Sandra Mazo (Católicas por el Derecho a Decidir - Colombia), Michelle Lopez (The National Latina Institute for Reproductive Justice), Deborah L Billings (University of South Carolina), Maria Julia Constant (Campaña por el Derecho al Aborto Legal, Seguro y Gratuito - Argentina), Kristen Baker (Feminist Center for Reproductive Liberation), and Maria M. Vivas Pérez (Fundación Oriéntame).”

The original version of this article has been updated.

